# Appropriate Management of Acute Diarrhea in Children Among Public and Private Providers in Gujarat, India: A Cross-Sectional Survey

**DOI:** 10.9745/GHSP-D-14-00209

**Published:** 2015-05-07

**Authors:** Christa L Fischer Walker, Sunita Taneja, Amnesty LeFevre, Robert E Black, Sarmila Mazumder

**Affiliations:** ^a^​ Johns Hopkins Bloomberg School of Public Health, Department of International Health, Baltimore, MD, USA.; ^b^​ Society for Applied Studies, Center for Health Research and Development, New Delhi, India.

## Abstract

Diarrhea remains a leading cause of morbidity and mortality among children under 5 years of age in low- and middle-income countries. In 2006, the Indian government formally endorsed the World Health Organization guidelines that introduced zinc supplementation and low-osmolarity oral rehydration salts (ORS) for the treatment of diarrhea. Despite this, zinc is rarely prescribed and has not been available in the public sector in India until very recently. The Diarrhea Alleviation Through Zinc and ORS Treatment (DAZT) project was implemented in Gujarat between 2011 and 2013 to accelerate the uptake of zinc and ORS among public and private providers in 6 rural districts. As part of an external evaluation of DAZT, we interviewed 619 randomly selected facility- and community-based public and private providers 2–3 months after a 1-day training event had been completed (or, in the case of private providers, after at least 1 drug-detailing visit by a pharmaceutical representative had occurred) and supplies were in place. The purpose of the interviews was to assess providers’ knowledge of appropriate treatment for diarrhea in children, reported treatment practices, and availability of drugs in stock. More than 80% of all providers interviewed reported they had received training or a drug-detailing visit on diarrheal treatment in the past 6 months. Most providers in all cadres (range, 68% to 100%) correctly described how to prepare ORS and nearly all (range, 90% to 100%) reported routinely prescribing it to treat diarrhea in children. Reported routine prescription of zinc was lower, ranging from 62% among private providers to 96% among auxiliary nurse-midwives. Among providers who reported ever *not* recommending zinc (n = 242), the 2 most frequently reported reasons for not doing so were not completely understanding zinc for diarrhea treatment and not having zinc in stock at the time of contact with the patient. In a multiple logistic regression analysis, recent training or drug-detailing visits and having zinc in stock were associated with reported zinc prescribing (*P*<.05). Recent training among public providers was significantly associated with having correct knowledge of zinc treatment duration and dosage, but the same was not true of drug-detailing visits among private providers. Treating diarrhea with zinc and low-osmolarity ORS is new for public and private providers in India and other low- and middle-income countries. Sufficient training and logistics support to ensure consistent supplies are critical if providers are to begin routinely treating all diarrhea episodes with zinc and ORS.

## Abstract (precis)

Training public-sector providers to treat diarrhea in children with low-osmolarity oral rehydration salts and zinc appeared to be effective. Among private providers, drug-detailing visits by pharmaceutical representatives seemed less effective, particularly in improving knowledge of the correct dosage and duration of zinc treatment. Consistent supplies and sufficient attention to training all health care cadres, especially community health workers who may be new to diarrhea treatment and informal-sector providers who are typically excluded from formal training, are critical to improving knowledge and prescribing behaviors.

## INTRODUCTION

Diarrhea is the second leading cause of death globally among children aged 1–59 months and accounts for 12.4% of deaths among all children under 5 years old in India.^1^ Diarrhea also causes substantial morbidity; children under 5 years old in India experience 2.4 episodes of diarrhea each year.^2^ Without prompt and correct treatment, frequent episodes can lead to stunting, which also contributes to cognitive delays.^3^^,^^4^

In 2004, the World Health Organization (WHO) released new recommendations for the treatment of diarrhea, including the liberal use of low-osmolarity oral rehydration salts (ORS) and zinc supplementation.^5^ The Indian Academy of Pediatrics endorsed these guidelines in 2004 and again in 2006.^6^^,^^7^ In 2007, the Ministry of Health and Family Welfare stated that zinc along with ORS should be available over-the-counter and in the public sector at the community health worker level–that is, among Accredited Social Health Activists (ASHAs) and Anganwadi workers (AWWs).^8^^,^^9^

Despite publication of these recommendations nearly 10 years ago, zinc and ORS are not widely used in India for the treatment of diarrhea. In many districts, availability of zinc remains scarce. Exact coverage has not yet been reported in state or national surveys. On the other hand, ORS has been widely available since the 1980s, but it is still not used in 74% of diarrhea cases.^10^

Little is known about the knowledge and prescription practices of public and private providers in India. In this article, we provide a brief description of a project implemented in 2 states of India that aimed to increase use of zinc and ORS to treat diarrhea in children by training public and private providers. We then report results of a survey of public and private providers’ knowledge and reported prescription practices after receiving the training.

## PROGRAM DESCRIPTION

The Diarrhea Alleviation Through Zinc and Oral Rehydration Salts Therapy (DAZT) project aimed to enhance the uptake of zinc and ORS among children 2–59 months of age over the course of 4 years in 12 districts of Uttar Pradesh and 6 districts of Gujarat in India. The main strategy of the project was to train public and private-sector providers on the appropriate use of zinc and ORS for diarrhea treatment.

### Public-Sector Activities

Micronutrient Initiative (MI), an international NGO, led the public-sector aspect of the project in all intervention districts. Public-sector activities focused on increasing the supply of zinc and ORS for diarrhea management and enhancing the diarrhea treatment capacity of all cadres of public-sector health care workers. To ensure the political and policy environment was primed for a public-sector campaign, MI collaborated with state and district governments to develop and promote appropriate diarrhea management policies and to establish a plan to routinely procure long-term zinc supplements. Initially, MI secured zinc and ORS products using its own tender scheme, to ensure the products were available in the intervention areas once training had started, until the state government was able to self-procure the products.

MI worked with the Ministry of Health in Gujarat and Uttar Pradesh to lead the training of all clinic and community health workers, including auxiliary nurse-midwives (ANMs), medical officers (MOs), ASHAs, and AWWs, on appropriate diarrhea management and the introduction of zinc treatment. MI first conducted a training of trainers, and the trainers subsequently trained groups of providers in each health worker cadre. The training continued until all providers in each project district had an opportunity to attend training. The training target was set at 100% of providers.

As part of the DAZT project, public-sector providers received training on appropriate management of diarrhea in children.

The 1-day training sessions were conducted in the local languages (Gujarati in Gujarat and Hindi in Uttar Pradesh) and covered:

- Signs and symptoms of diarrhea and dehydration
- Treatment of diarrhea, including how and why to prescribe zinc and ORS and the appropriate dose and duration of zinc and ORS
- Why to avoid unnecessary antibiotics
- When to refer diarrhea cases to higher-level health facilities

MOs and ANMs were trained together while community-based workers (ASHAs and AWWs) were trained in a separate group. The timing of the training sessions coincided with supply availability in each district so health care providers could start using their knowledge immediately.

### Private-Sector Activities

FHI 360, an international NGO, led the private-sector aspect of the DAZT project. The primary goals in the private sector were to increase the supply of zinc and ORS for diarrhea management, as in the public sector, and to provide necessary information to change the prescription practices among private providers in rural areas, including those in the formal sector (trained physicians), those in the informal sector (without formal medical degrees), and drug sellers/chemists.

FHI 360 worked with selected pharmaceutical companies and local NGOs to ensure quality zinc products were available in the targeted rural areas. To train physicians in the formal sector, FHI 360 conducted continuing medical education sessions on appropriate diarrheal management. Within each community, FHI 360 created lists of the most influential formal providers (i.e., eminent pediatricians and formal providers of the medical community) and then tracked and trained formal providers with smaller practices throughout the course of the project. For private providers in the informal sector and drug sellers/chemists, FHI 360 trained local NGO and pharmaceutical representatives to provide one-on-one drug-detailing visits to these providers, with the ultimate goal of repeat visits and thus ongoing drug-detailing visits throughout the program. During the short visits, the drug detailers distributed information, education, and communication (IEC) materials with basic information about treating diarrhea with zinc and ORS, and they also distributed the actual products themselves. Because rural providers in the informal sector often practice without formal training, this method enabled the project to access providers who did not have qualifications recognized by the government.

The DAZT project trained pharmaceutical representatives to provide supplies and information on zinc and ORS to private providers during drug-detailing visits.

The private-sector strategy was based on a cascade of knowledge from the formal to informal sector, and finally to the community to ensure demand creation and long-term sustainability. The project focused first on physicians with formal training because they have long-standing influence in their communities. Reaching informal providers, who are mostly not recognized by the government, was also important because they typically treat the majority of diarrhea patients in rural India. Additional details describing the implementation of the program and other aspects of the evaluation have been published elsewhere.^11^

## METHODS

The Johns Hopkins Bloomberg School of Public Health (Baltimore, Maryland) and the Society for Applied Studies (New Delhi, India) conducted an external evaluation of the DAZT project. The external evaluation team was not involved in program decision making or field implementation in any way. One component of the evaluation was a provider assessment survey conducted in 4 project districts in Gujarat state, which we present in this article. The survey was designed to capture the knowledge and reported zinc and ORS prescribing behaviors of providers 2–3 months after training had been completed and supplies were in place in the public sector and drug detailing was completed in the private sector. We interviewed private providers practicing in the targeted districts, as identified by FHI 360, and 4 cadres of public-sector providers: MOs, ANMs, ASHAs, and AWWs. During the interviews, we questioned providers about their knowledge of diarrhea treatment and attitudes toward prescribing zinc for diarrhea. We also assessed current stocks of zinc and ORS.

### Study Site

The study was conducted in Gujarat, which is home to more than 50 million people, including more than 4.7 million children under 5 years old.^12^ The DAZT project was implemented in 6 of the 26 districts of Gujarat, covering more than 10 million people, or 21.6% of the total population of Gujarat.^12^ The under-5 mortality rate in Gujarat was 56 per 1,000 live births in 2010,^12^ which was below the Indian national average of 61.3 per 1,000 live births.^1^ Until the start of the DAZT project, zinc had not been part of the routine treatment provided by either private or public-sector health care providers in Gujarat.

The provider assessment survey was conducted in 4 project districts (Banaskantha, Patan, Sabarkantha, and Surendranagar) in Gujarat, from December 2011 through January 2012. In the remaining 2 project districts, the public sector had not yet received products or training at the time of the provider assessment and were thus excluded from the assessment.

### Sample Size and Sampling Strategy

We calculated sample sizes for each group of providers separately. The sample sizes for ASHAs, AWWs, and private providers were based on anticipated zinc-prescribing of 10%, 10%, and 20%, respectively, and assumed a 10% margin of error, a design effect of 1.365,^10^ and a 15% to 20% increase for refusal to participate or for incomplete forms. We thus randomly selected 165 ASHAs, 165 AWWs, and 231 private providers to achieve a minimum of 140, 140, and 188 completed interviews, respectively. For MOs and ANMs, we calculated a Lot Quality Assurance Sample (LQAS) and interviewed 1 MO and 2 ANMs per primary health center (PHC).^13^

We included public-sector providers from 33 of the 236 PHCs in the 4 districts of Gujarat. The number of PHCs sampled per district was determined using a probability proportional to size (PPS) sampling design, such that the number of PHCs selected was based on the proportion of PHCs in that district to overall PHCs in all districts. We used STATA 12.0 to generate a random sample of PHCs from within the district. We obtained a list of all MOs, ANMs, ASHAs, and AWWs at each PHC in advance. At each PHC, 5 ASHAs, 5 AWWs, 2 ANMs, and 1 MO were selected for inclusion in the study. There were no additional inclusion/exclusion criteria for the providers other than actively practicing as an ASHA, AWW, ANM, or MO. For MOs and ANMs, there were often not more than 1 MO and 2 ANMs from which to choose; if more than 1 MO or 2 ANMs were practicing at the selected PHC, the names were randomly selected. The names of all active ASHAs were collected in advance, and 5 ASHAs were randomly selected from each PHC. In order to aid in the logistics of conducting the survey, we selected the AWW posted closest to the randomly selected ASHA. In the case of MOs and ANMs, if not enough MOs or ANMs were present at the PHC, an additional PHC was visited from the same district to ensure the entire samples size was achieved. For ASHAs and AWWs, the health workers were identified randomly in advance; if the identified health worker was not available during the 2 days of interviews at each PHC, the next ASHA/AWW from the randomization list was included in the survey.

At the time of the provider assessment, FHI 360 had collected the names of 1,337 private providers working in the 4 target districts included in this survey. Private providers were randomly selected in advance from the corresponding villages serviced by the randomly selected PHCs; we selected 7 private providers per PHC area. Locating specific private providers in this informal sector was often challenging. We created a system of asking at least 3 community leaders for the location of the identified provider before accepting his/her absence and selecting the next private provider from the randomization list. If the private provider was not located within 48 hours of the desired interview time or after 3 village leaders confirmed that the private provider did not exist or was no longer present, the private provider was replaced with the next private provider on the randomization list. If the sample size of private providers could not be met in the selected villages, the next randomly selected PHC area and the corresponding private providers were chosen from within the corresponding villages.

### Survey Methods

We trained all interviewers using a standard protocol and implemented the survey 2–3 months after completion of training to minimize knowledge loss. Interviewers obtained written informed consent and administered closed-ended survey questionnaires to selected providers. The survey lasted approximately 1 hour and included questions on diarrhea treatment knowledge and practices, as well as access to routinely available ORS and zinc supplies. All questions on knowledge were asked before questions on typical practice. To ensure confidentiality, all interviews took place in private locations (i.e., in the clinic, health facility, or home of community-based workers). All data were checked in the field and inconsistencies/incomplete forms were corrected immediately by returning to the provider for verification before the team moved to another PHC. Data were entered twice (double data entry) into a standardized data entry system; queries and inconsistencies were verified by calling the provider if necessary.

### Ethical Approval

We received ethical approval for this study by both the Johns Hopkins Bloomberg School of Public Health Institutional Review Board and the Society for Applied Studies Ethical Review Board. All providers were informed about the risks and benefits of the study, and they signed consent forms before participating in the survey. We also obtained permission from the Government of Gujarat to interview public-sector health workers before starting the survey.

### Analytic Methods

We calculated means and standard deviations, medians, and corresponding ranges, as well as proportions for selected characteristics of ANMs, MOs, ASHAs, AWWs, and private providers. We assessed knowledge and reported practices among ASHAs, AWWs, and private providers and calculated the proportions who reported correct responses for key knowledge and practice indicators. We performed a chi-squared test for multiple comparisons to determine if there were any statistically significant differences in responses across provider types. We then conducted a multiple logistic regression to determine if provider education, experience, training/drug-detailing visits, and product availability were independently associated with provider prescribing practices or knowledge of dose, duration, and/or ORS preparation for ASHAs, AWWs, and private providers. All statistical tests were conducted using STATA 12.0 Statistical Software.^14^ For ANMs and MOs, we calculated the proportion of providers who reported correct responses for key knowledge and practice indicators.

## RESULTS

**FIGURE 1.**
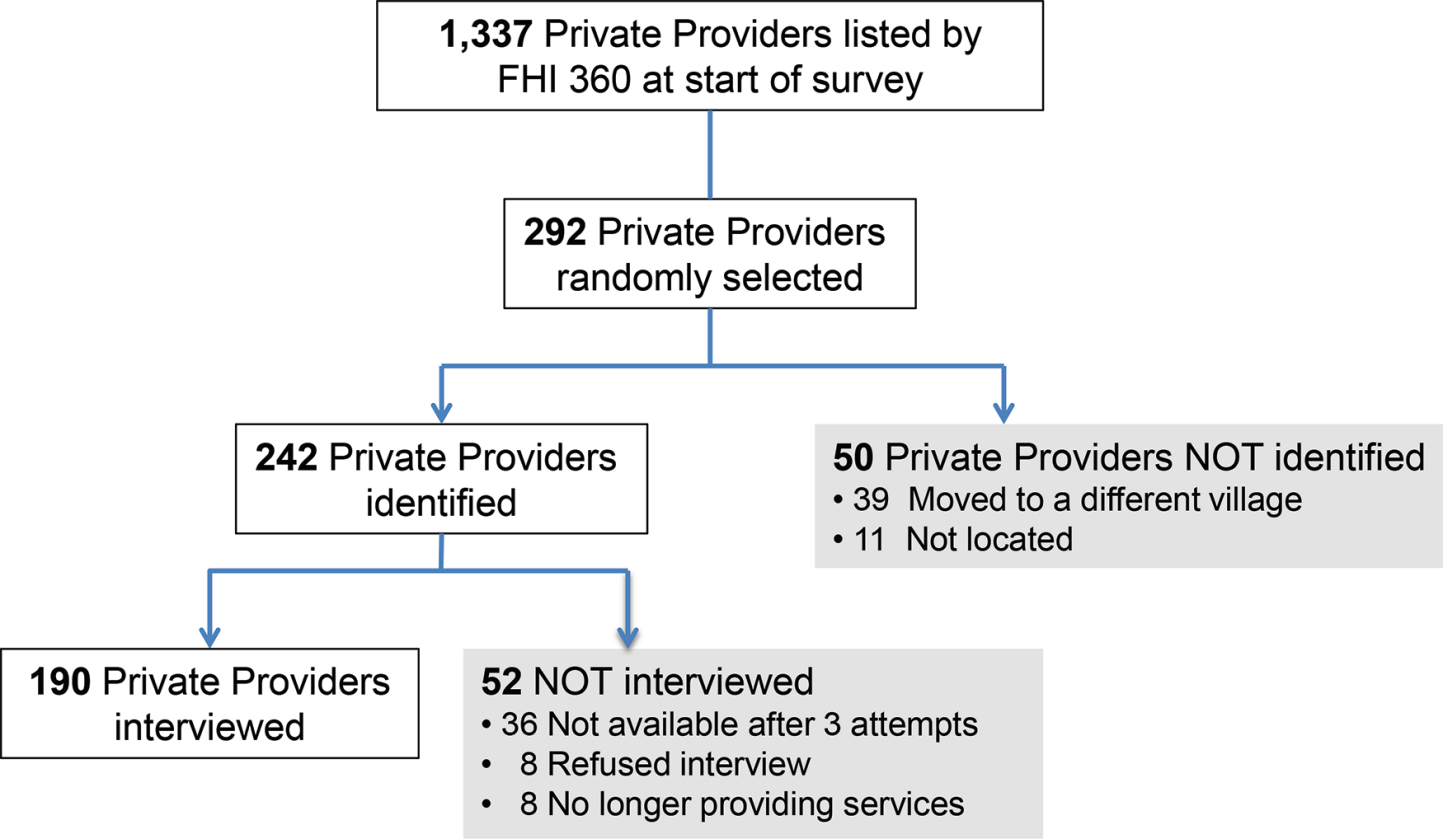
Flowchart of Private Provider Survey Participants

**FIGURE 2.**
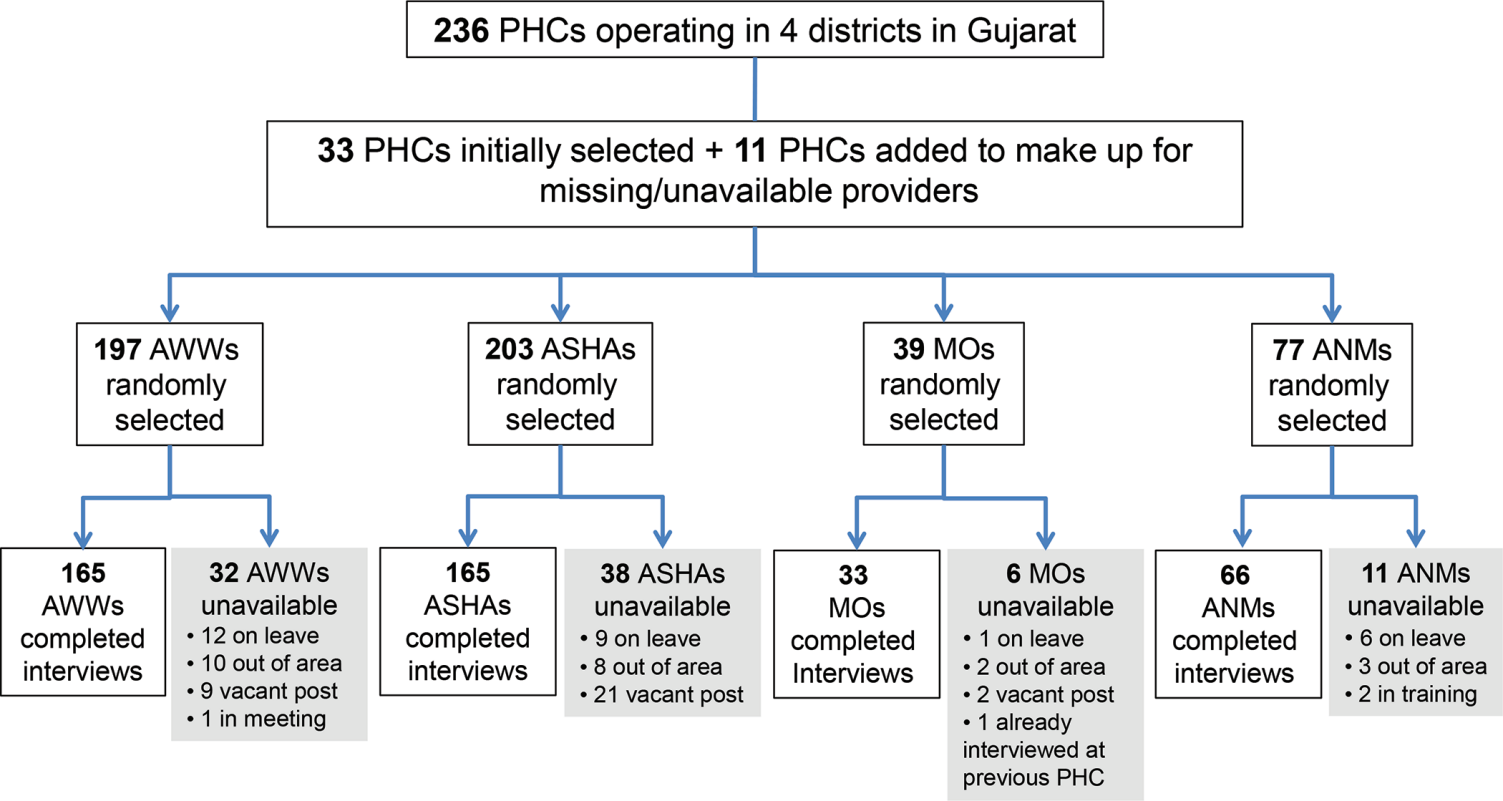
Flowchart of Public Provider Participants Abbreviations: ANMs, auxiliary nurse-midwives; ASHAs, Accredited Social Health Activists; AWWs, Anganwadi workers; MOs, medical officers; PHCs, primary health centers.

We interviewed in total 619 providers: 190 private providers (Figure 1), 165 AWWs, 165 ASHAs, 33 MOs, and 66 ANMs (Figure 2), achieving our desired sample size for each provider category. To achieve the minimum sample size, we had to include providers from 11 additional PHCs/villages (for the community-based workers). While every effort was made to find and interview the provider selected according to the random selection procedure *a priori*, in some cases the individual selected could not be found or too few providers of that provider type were available and working at the selected PHC. For example, to achieve the minimum sample size among private providers, we initially randomly selected 231 providers and then randomly selected an additional 61 providers during a second round, which led to 190 (65%) completed interviews.

All interviewed ANMs, ASHAs, and AWWs were women while nearly all the private providers (97%) and MOs (82%) were men (Table 1). Community health workers (ASHAs and AWWs) had a median of 10 years of education, and ANMs and private providers had a median of 12 and 16 years of education, respectively. (All MOs were trained physicians who had passed their medical examinations successfully.) Most providers (around 83% to 88%) reported receiving training/drug-detailing visits in the treatment of diarrhea within the last 6 months. Higher proportions of providers of all cadres had ORS than zinc in stock. ANMs were most likely to report zinc in stock (66.7%) while MOs were most likely to report ORS in stock (93.9%); private providers were least likely to report zinc or ORS in stock (36.3% and 55.3%, respectively).

More providers reported they had ORS in stock than zinc.

**TABLE 1.** Characteristics of Health Care Providers in 4 Districts of Gujarat, India

	**ASHAs (n=165)**	**AWWs (n=165)**	**ANMs (n=66)**	**MOs (n=33)**	**PPs (n=190)**
Age, mean (SD), y	29.4 (6.4)	39 (8.2)	39.6 (9.3)	34.3 (7.4)	43.2 (13.8)
Female, No. (%)	165 (100)	165 (100)	66 (100)	6 (18.2)	5 (2.6)
Years of education, mean (range)	10 (4, 17)	10 (4, 17)	12 (10, 19)	NA^a^	16 (9, 22)
Years practicing in current position, median (range)	3 (1, 18)	13 (1, 32)	19 (1, 28)	3 (1,24)	10 (1, 45)
Providers who have received training/drug-detailing visits for diarrhea treatment in last 6 months, No. (%)	138 (83.6)	144 (87.3)	55 (83.3)	29 (87.9)	168 (88.4)
Providers who had any zinc in stock, No. (%)	93 (56.4)	81 (49.1)	42 (66.7)	21 (63.6)	69 (36.3)
Providers who had any ORS in stock, No. (%)	108 (65.5)	94 (57.0)	51 (77.3)	31 (93.9)	105 (55.3)

Abbreviations: ASHAs, Accredited Social Health Activists; AWWs, Anganwadi workers; ANMs, accredited nurse-midwives; MOs, medical officers; ORS, oral rehydration salts; PPs, private providers; SD, standard deviation.

a All MOs were trained physicians who had passed their medical examinations successfully.

Most providers answered correctly that children with diarrhea should increase fluid intake, with the highest level of correct knowledge among private providers (77%) (Table 2). Few or no providers in any cadre thought that children with diarrhea should decrease fluid intake. Most providers also answered correctly that children with diarrhea should maintain or increase their food intake, but a smaller proportion of private providers knew this compared with the other cadres (72% of private providers vs. 85% of MOs and about 90% of ASHAs, AWWs, and ANMs). High percentages (85% or higher) of all provider types could recall at least 2 signs or symptoms requiring referral, but much lower proportions (15% to 39%) could name 4 or more signs or symptoms.

Most providers knew that children with diarrhea should increase fluid intake.

**TABLE 2.** Diarrhea Management Knowledge and Reported Prescribing Behaviors Among Public and Private Providers, Gujarat, India

	**Public-Sector Providers**				
	**ASHAs (n=165)**	**AWWs (n=165)**	**ANMs (n=66)**	**MOs (n=33)**	**PPs (n=190)**
No. (%) reporting a child with diarrhea should:					
Increase fluid intake* *(correct response)*	106 (64.2)	118 (71.5)	49 (74.2)	24 (72.7)	147 (77.4)
Maintain usual fluid intake	48 (29.1)	40 (24.2)	17 (25.8)	9 (27.3)	40 (21.1)
Reduce fluid intake*	11 (6.7)	7 (4.2)	0 (0)	0 (0)	3 (1.6)
Maintain usual or increase breastfeeding/food intake* *(correct response)*	146 (88.5)	150 (90.1)	59 (89.3)	28 (84.8)	136 (71.6)
Reduce food intake*	19 (11.5)	15 (9.1)	7 (10.6)	5 (15.2)	54 (28.4)
No. (%) recalling ≥ 2 signs/symptoms requiring referral to higher-level facility*^a^	141 (85.5)	154 (93.3)	65 (98.5)	30 (90.9)	162 (85.3)
No. (%) recalling ≥ 4 signs/symptoms requiring referral to higher-level facility*^a^	40 (24.2)	56 (33.9)	26 (39.4)	5 (15.2)	44 (23.2)
No. (%) reporting *usually* recommending zinc treatment*	137 (83.0)	146 (88.5)	63 (95.5)	29 (87.9)	117 (61.6)
No. (%) correctly stating the dose and duration of zinc syrup or tablet					
Duration of 14 days*	140 (85.0)	144 (87.0)	63 (95)	31 (94)	90 (47.0)
Correct dose for children 2–5 months old (10 mg/day, i.e., 1/2 tablet or 5 mL)*^b^	60 (36.0)	53 (32.0)	47 (71)	20 (61)	33 (17.0)
Correct dose for children 6–59 months old (20 mg/day, i.e., 1 tablet or 10 mL)*^b^	88 (53.0)	84 (51.0)	49 (74)	22 (67)	47 (25.0)
Refused to answer/did not know correct dose^a^	18 (11.0)	10 (6.0)	0 (0)	0 (0)	146 (77.0)
No. (%) reporting *routinely* recommending ORS*	164 (99.4)	164 (99.4)	66 (100)	32 (97.0)	170 (89.5)
No. (%) correctly describing how to prepare ORS *(i.e., 1 L packet in 1 L water or 200 mL packet in 1 cup of water)* *	157 (95.7)	153 (93.3)	62 (93.9)	33 (100)	114 (67.5)

Abbreviations: ANMs, accredited nurse-midwives; ASHAs, Accredited Social Health Activists; AWWs, Anganwadi workers; MOs, medical officers; ORS, oral rehydration salts; PPs, private providers.

* Signifies differences in response by provider type using chi-squared test for multiple proportions (*P*<.05).

a Signs/symptoms included: unconscious, lethargic, convulsions, unable to drink or breastfeed, persistent diarrhea, sunken eyes, skin pinch goes back slowly, irritable/restless, blood in stool, signs of mild dehydration, fast breathing, difficulty breathing, and vomiting.

b Government and training documents state infant dosing is from 2 months and up to 6 months of age. We considered 2–5 months and 2–6 months as correct responses. For this reason, we also accepted 6 or 7 months as the lower bound of the older age category. Zinc should be given until 5 years of age, and thus 59 or 60 months were accepted as the upper bound for the older age category.

**FIGURE 3.**
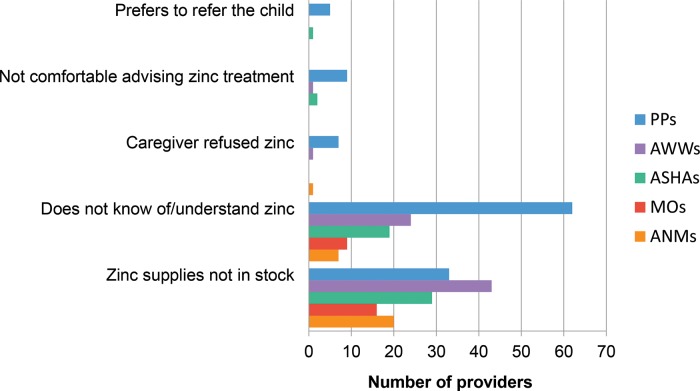
Main Reasons^a^ for Not Recommending Zinc Among Providers Who Reported Ever Not Recommending Zinc, Gujarat, India (n=242)^b^ Abbreviations: ANMs, auxiliary nurse-midwives; ASHAs, Accredited Social Health Activists; AWWs, Anganwadi workers; MOs, medical officers; PPs, private providers. ^a^ Other reasons (not shown on chart) reported by private providers but no other provider cadre: caregiver could not afford zinc (17); caregivers prefer treatments that provide quick recovery (7); other drugs are better for diarrhea treatment (4); zinc is not widely accepted among providers (3); profit margin for zinc is not as large as for other drugs (2); zinc is not an effective treatment (2). ^b^ The 242 providers who reported ever not recommending zinc included 107 private providers, 56 AWWs, 38 ASHAs, 19 MOs, and 22 ANMs.

The percentage of providers stating they usually prescribed zinc for childhood diarrhea ranged from only 62% of private providers to 96% of ANMs. Among providers who reported ever *not* recommending zinc (n = 242), we asked unprompted questions about why. The two most frequent responses were not knowing of or completely understanding zinc for diarrhea treatment and not having zinc in stock at the time of contact with the patient (Figure 3).

The most commonly reported reasons for not prescribing zinc were lack of knowledge about zinc for diarrhea treatment and not having zinc in stock.

Among public-sector providers, 85% or higher recalled the correct duration of zinc treatment (14 days), but much lower percentages recalled the correct dosage (Table 2). For example, only 36% and 53% of ASHAs stated the correct dosage for children 2–5 months and 6–59 months of age, respectively. Among private providers, only 47% stated the correct duration of zinc treatment and 77% did not know or refused to answer when asked about the correct dose of zinc.

Much higher percentages of providers of all cadres had correct knowledge of ORS and reported routinely prescribing ORS compared with zinc. In fact, reported prescribing practices for ORS were nearly universal, ranging from 90% of private providers to 100% of ANMs. Nearly all public-sector providers correctly described how to prepare ORS (93% or higher among public-sector provider cadres), but only 68% of private providers described the process correctly.

In multiple logistic regression analysis of factors predicting public-sector provider knowledge and prescribing patterns, receiving training in diarrhea treatment within the last 6 months was predictive of routinely recommending zinc treatment and of generally having correct knowledge of zinc treatment duration and dosage among ASHAs and AWWs (*P*< .01) (Table 3). Similarly, among private providers, receiving drug-detailing visits (and CME training, in the case of formal-sector providers) for diarrhea treatment within the last 6 months was predictive of recommending zinc treatment (*P* = .01) but not for having correct knowledge of the duration or dosage of zinc. ASHAs and private providers who had zinc in stock were also more likely to report routinely prescribing zinc than their counterparts who did not have zinc in stock (odds ratio [OR] =  4.98 and 7.00, respectively). Among private providers, years of education (OR = 1.44), receiving a recent drug-detailing visit (OR = 3.86), and having ORS in stock (OR = 4.12) were all predictive of the provider reporting routinely prescribing ORS. The regression model for the outcome of reported routine ORS prescribing was invalid among ASHAs and AWWs because more than 99% of these providers reported routinely recommending ORS.

Recent training or drug-detailing visits was significantly associated with providers reporting regularly prescribing zinc to treat diarrhea.

**TABLE 3.** Demographic and Training Variables Associated With Knowledge and Reported Practice Indicators for Diarrhea Treatment With Zinc and ORS by Type of Provider,^a^ Gujarat, India

**Demographic and Training Variables**	**Knowledge and Reported Practice Outcome Variables**											
	**Reported routinely recommending zinc**		**Knew correct duration of zinc treatment**		**Knew correct zinc dose for 2–5- month-old child**		**Knew correct zinc dose for 6–59- month-old child**		**Reported routinely recommending ORS**		**Knew correct ORS preparation**	
	**OR**	*****P*** value**	**OR**	*****P*** value**	**OR**	*****P*** value**	**OR**	*****P*** value**	**OR**	*****P*** value**	**OR**	*****P*** value**
**ASHAs (n = 165)**												
Years of education	0.88	.25	1.05	.68	1.01	.94	1.03	.65	NA^b^		1.28	.27
Years practicing as ASHA	0.87	.13	0.92	.40	1.00	.59	0.94	.45	NA^b^		**2.60**	**.02**
Received training in diarrhea treatment in last 6 months	**9.04**	**<.001**	**4.85**	**<.001**	**3.45**	**.04**	**4.80**	**<.001**	NA^b^		1	NA
Zinc in stock	**4.98**	**.02**	**7.20**	**.01**	0.00	.99	**3.52**	**.05**	NA^b^		7.10	.09
ORS in stock	0.38	.22	0.73	.64	0.00	.99	0.37	.14	NA^b^		0.15	.13
**AWWs (n = 165)**												
Years of education	0.93	.58	1.04	.73	1.04	.56	1.03	.65	NA^b^		1.14	.35
Years practicing as AWW	1.01	.71	1.00	.94	1.03	.17	0.99	.82	NA^b^		1.10	.06
Received training in diarrhea treatment in last 6 months	**16.05**	**<.001**	**5.68**	**<.001**	2.80	.13	**4.85**	**.01**	NA^b^		1.51	.61
Zinc in stock	1.94	.48	**34.82**	**<.001**	2.16	.36	1.18	.81	NA^b^		**6.84**	**.05**
ORS in stock	0.72	.70	**0.15**	**.01**	0.47	.37	1.21	.78	NA^b^		0.27	.13
**PPs (n = 190)**												
Years of education	**1.24**	**<.001**	**1.20**	**<.001**	1.17	.06	**1.16**	**.04**	**1.44**	**<.001**	0.98	.72
Years practicing as PP	1.01	.57	0.99	.68	1.01	.47	1.00	.86	1.02	.42	0.98	.18
Received drug-detailing visit for diarrhea medications in last 6 months	**3.95**	**.01**	1.96	.21	3.77	.21	2.79	.19	**3.86**	**.04**	0.55	.25
Zinc in stock	**7.00**	**<.001**	**2.51**	**.01**	1.98	.12	**2.29**	**.03**	5.55	.12	1.82	.10
ORS in stock	0.57	.14	0.99	.98	1.05	.91	0.84	.64	**4.12**	**.03**	1.11	.75

Abbreviations: ASHAs, Accredited Social Health Activists; AWWs, Anganwadi workers; NA, not applicable; OR, odds ratio; ORS, oral rehydration salts; PPs, private providers; RMP, rural medical practitioner.

a Results are from a multiple logistic regression (MLR) analysis. Statistically significant results at *P*≤ .05 are shown in boldface.

b Not applicable in the MLR analysis because 99.4% of ASHAs and AWWs reported routinely recommending ORS for the treatment of diarrhea.

## DISCUSSION

In our survey of public and private providers’ knowledge and reported prescribing practices for treatment of childhood diarrhea, nearly all providers in all cadres correctly described how to prepare ORS (except for private providers, for whom only 68% had such correct knowledge) and reported routinely prescribing ORS to treat diarrhea in children. In contrast, knowledge of the correct duration, and particularly of the correct dosage, of zinc treatment for diarrhea was generally lower among provider cadres. Public-sector providers who had received recent training were more likely to have correct zinc knowledge and reported prescribing practices while private providers who had received recent drug-detailing visits in diarrhea treatment, or recent CME training in the case of formal-sector providers, were more likely to report prescribing zinc to treat diarrhea but not to have correct knowledge of the duration or dosage of zinc.

Correct knowledge of ORS preparation was generally higher than of zinc duration or dosage among all provider cadres.

The results of this cross-sectional survey represent an initial point in time by which all public-sector providers should have received training and all private-sector providers should have received at least 1 drug-detailing visit. The DAZT project is responsible for reaching scale in these 4 districts, which cover a population of more than 7 million people. This is one of the first surveys to include both public and private-sector providers in a diarrhea treatment program of this scale.

In rural Gujarat, there are many types of private providers loosely categorized as rural medical practitioners including those with little to no formal education,^14^ yet in our survey 76% of private providers had education beyond grade 12. The higher education level of private providers in our survey may represent the early stages of the project’s private-sector implementation strategy, which focused on reaching providers who were key opinion leaders; such providers tended to be pediatricians and formal medical providers or very well trained providers in the informal sector. The project expanded to harder-to-reach providers, who were likely less educated, later in the program.

The survey found much confusion among providers about the correct dose and duration of zinc. While public-sector training did address zinc dosage and duration, further on-the-job training and distribution of job aids are probably warranted, especially for community health workers (ASHAs and AWWs). Knowledge of the correct dose and duration of zinc was particularly poor among private providers; more than 75% refused to answer the question about zinc dosage or admitted to not knowing the answer. The pharmaceutical package of IEC materials for zinc provided information on dose and duration of treatment, but it may have been overlooked during the detailing visit as a minor detail, especially if time for the visit was cut short. In addition, there are many brands of zinc in both tablet and syrup formulas available in the private sector, which may create an additional level of uncertainty among private providers. Formal training of private providers is probably needed, rather than depending solely on drug detailing by pharmaceutical company representatives.

Formal training of private providers on appropriate treatment of diarrhea, instead of relying solely on drug-detailing visits, is probably needed.

The DAZT project did not implement demand-side interventions such as community education or social marketing activities. All changes in reported prescribing practices were thus driven by introducing zinc and reinforcing ORS to the providers. Drug detailing among private providers focused on the positive elements of zinc and ORS only, without specifically addressing the unnecessary use of antibiotics. A high proportion of private providers reported antibiotics as appropriate treatments for simple acute watery diarrhea (data not shown), highlighting the need for training specifically on this issue. As mentioned above, an alternative approach besides drug detailing by pharmaceutical companies, that have profits in mind and may not want to discuss the potential harm of antibiotics, will be needed to address the issue of antibiotics. This is especially challenging because in many countries private providers often do not have formal training and are not recognized by the government as legitimate health care providers.

### Limitations

In this survey, we interviewed randomly selected providers and emphasized that the interview team was in no way associated with the government or any regulating body. Interviewers encouraged the interviewees to answer questions openly and honestly and reminded them that their answers would not be revealed to supervisors. Despite this, providers may have been swayed to answer practice questions with a known “right” answer even if it did not accurately reflect their true practices. It is possible that by notifying PHCs and MOs in advance of our visit, active brief refresher trainings could have been initiated, although the advance warning was only 1–2 days so it would not have been possible to do anything more than minimal preparation. Additional insights from direct observation would validate and/or challenge the responses provided in this survey.

In addition, drawing the sample of private providers from the same communities captured in the sample of public-sector providers may have reduced apparent reported differences between public and private providers. Furthermore, we are limited in the interpretation of the survey results and attribution of correct knowledge and reported prescribing practices to the DAZT project without a baseline survey to measure change over time. However, because there had been no prior training or drug detailing with regard to zinc in either the public or the private sector, it is likely that zinc knowledge was very limited prior to this program. It also would have been interesting to conduct a survey at the end of the project to measure change in knowledge and reported practices after the providers had had more experience with prescribing zinc and ORS but not necessarily additional opportunities for continued training. Finally, failure to identify all originally selected providers creates possible bias in that we were more likely to exclude harder-to-find providers or those who may not be available to treat patients full time—also the same kinds of providers who would not be present for training opportunities.

## CONCLUSIONS

With training about appropriate treatment of childhood diarrhea with both zinc and ORS and access to the products, some providers report prescribing zinc, but long-term sustained use and comfort with the new recommendations will require additional training and distribution of job aids, and possibly other behavior change approaches, to help providers overcome early skepticism and increase familiarization with the correct dose and duration. Overcoming inconsistencies with the supply chain will also be critical to create an early habit of prescription for both community-level workers who are new to community case management, such as ASHAs and AWWs, and private providers who have a history of relying on inappropriate use of antibiotics and antidiarrheals for the treatment of all diarrhea episodes. While the focus of the DAZT project was on improving knowledge and use of zinc and ORS among providers, community-level introduction and public awareness of zinc and ORS for diarrhea treatment could generate a demand-side pull that might also promote correct prescribing practices from both public and private providers. Health systems will need to monitor how barriers to prescribing zinc and ORS change as diarrhea management programs mature and communities become more familiar with new treatment protocols in both the public and the private sectors.

## Notes

**Peer Reviewed**
